# [Retracted] MicroRNA-202 inhibits endometrial stromal cell migration and invasion by suppressing the K-Ras/Raf1/MEK/ERK signaling pathway

**DOI:** 10.3892/ijmm.2024.5375

**Published:** 2024-04-08

**Authors:** Di Zhang, Ling Wang, Hua-Lei Guo, Zi-Wei Zhang, Chong Wang, Ri-Cheng Chian, Zhi-Fen Zhang

Int J Mol Med 46: 2078-2088, 2020; DOI: 10.3892/ijmm.2020.4749

Following the publication of this paper, it was drawn to the Editor's attention by a concerned reader that certain of the Transwell invasion assay data shown in Figs. 2C and 4B were strikingly similar to data appearing in different form in a paper by different authors at a different research institute that had already been submitted for publication.

Owing to the fact that the contentious data in the above article had already been submitted for publication prior to its submission to *International Journal of Molecular Medicine*, the Editor has decided that this paper should be retracted from the Journal. The authors were asked for an explanation to account for these concerns, but the Editorial Office did not receive a satisfactory reply. The Editor apologizes to the readership for any inconvenience caused.

